# "Double Frozen Transfer" Could Influence the Perinatal and Children's Growth: A Nested Case-Control Study of 6705 Live Birth Cycles

**DOI:** 10.3389/fendo.2022.878929

**Published:** 2022-08-12

**Authors:** Jie Gao, Yiyuan Zhang, Linlin Cui, Tao Zhang, Bingjie Wu, Shanshan Gao, Zi-Jiang Chen

**Affiliations:** ^1^Center for Reproductive Medicine, The Second Hospital, Cheeloo College of Medicine, Shandong University, Jinan, China; ^2^Research Unit of Gametogenesis and Health of ART-Offspring, Chinese Academy of Medical Sciences, Jinan, China; ^3^Key laboratory for Reproductive Endocrinology, Ministry of Education, Shandong University, Jinan, China; ^4^Shandong Provincial Clinical Medicine Research Center for Reproductive Health, Jinan, China; ^5^Department of Biostatistics, School of Public Health, Shandong University, Jinan, China; ^6^Center for Reproductive Medicine, Renji Hospital, Shanghai JiaoTong University School of Medicine, Shanghai, China; ^7^Shanghai Key Laboratory for Assisted Reproduction and Reproductive Genetics, Shanghai, China

**Keywords:** frozen embryo transfer (FET), double frozen transfer, gamete cryopreservation, fresh embryo transfer (ET), neonatal outcome, children growth

## Abstract

**Objective:**

This study aims to evaluate neonatal and children growth outcomes of cryotransfer of embryos developed from frozen gametes [double frozen transfer (DFT)].

**Methods:**

This nested case-control study included 6,705 women who had a singleton live birth after embryo transfer at the Center for Reproductive Medicine, Shandong University, from 2008 to 2020. Of these, 745 women underwent frozen embryo transfer (FET) using embryos developed from frozen gametes (DFT). Propensity score methodology was used to balance the two groups by maternal age and body mass index (BMI) before evaluating outcomes. After age and BMI were matched using the propensity score methodology in a ratio of 1:4, the control groups enrolled 2,980 women who underwent fresh embryo transfer (ET) and 2,980 women underwent FET from fresh gametes. The children born were followed to at least 5 years of age, and some were followed up to 10 years. Neonatal outcomes and childhood growth measurements were compared among the three groups.

**Results:**

The average birth weight of the DFT group (3,462 g) was significantly higher than the FET group (3,458 g) and ET group (3,412 g). The rate of large for gestational age (LGA) babies in the DFT and FET group was higher than that for the ET group (30.9% vs. 24.8%; 29.4% vs. 24.8%, respectively). After adjusting for different confounder combinations in the three models, the birth weight and risk of LGA in the DFT and FET groups were still higher than in the ET group, and the values group of *P* for trend in the models were significant. In multiple linear regression analysis of the children’s development, the height Z-score of children born from the DFT and FET group was higher than that for children from the ET group (β = 0.21, 95% CI 0.07–0.35; b = 0.17, 95% CI 0.05–0.28, respectively). However, childhood growth measurements including body weight Z-score and BMI Z-score were not significantly different among the three groups. In addition, the proportion of male children born from DET was higher than that from ET.

**Conclusions:**

There is an increased risk of LGA babies associated with pregnancies conceived from DFT. Children are inclined to be taller in the future in this group than after FET. The related etiology and pathophysiology mechanisms still need to be revealed. In the future, well-designed, observational studies with in-depth collection of patients’ characteristics may shed more light on this issue.

## Introduction

Worldwide, more than eight million children have been conceived after assisted reproductive technology (ART) (1). However, studies have shown that pregnancies and deliveries resulting from ART are generally associated with adverse obstetric and perinatal outcomes when compared to spontaneously conceived (SC) pregnancies (2, 3). Concerns about the safety of ART are increasing, and frozen embryo transfer (FET) and gamete cryopreservation, as important components of ART, have recently focused on perinatal and neonatal outcomes (4, 5).

Literature shows that FET is related to a decrease in the incidence of low birth weight, small for gestational age (SGA), preterm birth, placenta previa, and placental abruption compared with fresh embryo transfer (ET). However, evidence from two recent meta-analyses shows some adverse obstetrics and perinatal outcomes after FET including pregnancy-induced hypertension (PIH), large for gestational age (LGA), and postpartum hemorrhage (4, 6). There are also studies on the perinatal and neonatal outcomes from gamete cryopreservation applied in ART. Most cohort studies show no increased risk of adverse perinatal outcomes following donor sperm compared with partner sperm *in vitro* fertilization (IVF)/intracytoplasmic sperm injection (ICSI) treatment (7–9). A recent systematic review regarding the impact of oocyte vitrification on offspring shows that vitrification seems to be a safe method for oocyte cryopreservation and child health, at least in the short term (10).

However, to the best of our knowledge, almost no studies have focused on the obstetric and offspring outcome from the cryotransfer of embryos developed from frozen gametes. It is interesting to speculate that there may be an accumulating effect on the offspring after double frozen transfer (DFT). The present study aimed to evaluate the effect of DFT on the outcomes of neonatal and children growth by comparing it with “single frozen transfer” FET and fresh ET in a nested case-control study.

## Materials and Methods

### Study Design and Oversight

To determine whether double freezing-thawing procedures influence the short- and long-term health of offspring, we assess perinatal and neonatal outcome along with children growth of DFTs, FETs, and ETs. We conducted a single-center, retrospective, nested case-control analysis at the Center for Reproductive Medicine Affiliated to Shandong University. The study was approved by the Institutional Review Board of the Second Hospital, Cheeloo College of Medicine, Shandong University. The ethics approval document number is 2022(37). Written informed consent was obtained from patients and parents or guardians of all participants.

### Study Population

This was a matched case-control retrospect analysis including DFT, FET and ET during April 2008 and May 2020 (**Supplementary Figure 1**). A total of 6705 patients were included in this study. The 745 patients in the DFT group underwent cryotransfer of embryos developed from frozen gametes; of which, 721 cycles involved sperm cryopreservation and 24 cycles involved oocyte vitrification. After maternal age and body mass index (BMI) were matched with the propensity score methodology in a ratio of 1:4, the control group enrolled 2980 cycles that underwent FET and 2980 cycles that underwent ET. All embryos transferred resulted in a singleton birth. Children were followed from birth to at least 5 years to assess growth information including height, weight, and BMI; some children were followed to 10 years of age. Patients were excluded if they were multi-gestation or had been delivered before 28 weeks of pregnancy or stillbirth.

### Study Procedures

After routine ovarian stimulation protocols, as previously described (11, 12), transvaginal ultrasound-guided oocyte retrieval was carried out 34–38 h after Human chorionic gonadotropin (HCG) administration. Oocyte fertilization was achieved by IVF or ICSI based on the male partner’s sperm quality. High-quality embryos were selected for transfer at the cleavage-stage or blastocyst stage, and a maximum of two embryos were transferred. Fresh embryos are preferentially transferred at cleavage stage, whereas FET tends to transfer blastocyst embryos. Surplus or all blastocysts were vitrified on day 5 or day 6, based on embryo development, for future transfer. Sperm cryopreservation was used in two situations: the first was autologous sperm cryopreservation as a backup sperm source and the second was cryopreserved donor semen. Oocyte vitrification was used in clinical scenarios such as the unavailability of sperm at the time of egg retrieval or for couples who did not wish to cryopreserve supernumerary embryos in cases where plenty of oocytes were retrieved. Another indication for oocyte vitrification that has now become a reality is the establishment of donor oocyte banks.

### Pregnancy Assessment and Follow-Up

Endometrial preparation for FET is described in detail elsewhere (13). Luteal support continued until 11–12 weeks of gestation. Clinical pregnancy was determined through transvaginal ultrasonography by detecting one or more gestational sacs. Early miscarriage was defined as the spontaneous loss of clinical pregnancy within the first 13 weeks of gestation. Subsequently, each patient would receive a telephone survey and standardized questionnaires delivered by trained nurses. Information would be collected including perinatal complications, gestational weeks, birth date, delivery mode, newborn gender and birth weight, neonatal diseases, treatment, and prognosis. All follow-up information was recorded in the electronic medical records (14).

A live birth was defined as the delivery of a viable infant after 28 weeks of gestational age. Low birth weight was defined as a newborn baby weighing below 2,500 g. small for gestational age (SGA) was defined as a birth weight below the 10th percentile for gender and gestational age according to the reference population. Birth weight for gender- and gestational age–specific standard score (z-score) was calculated on the basis of a Chinese reference chart (15). Z-score was calculated according to the following formula: (weight of an individual infant at a given gestational age − mean weight of the reference population at the same gestational age)/standard deviation (SD) in the reference population. Pediatric growth parameters included height in centimeters, weight (kg), and BMI (kg/m^2^). Data recorded also included whether or not the infant was breastfed.

### Statistical Methods

All data analyses were performed using SPSS statistical software v26 and R v4.0.2. Propensity score matching was used to balance the baseline maternal characteristics among the three groups. Patients of DFT, FET and ET groups were evaluated using the propensity score methodology with nearest neighbor matching (caliper 0.2). The matching ratio was 1:4 with the matching factors referring to maternal age and BMI.

Confounders were enrolled according to clinical experience and up-to-date literatures. Continuous variables were presented as mean ± standard deviation with one-way analysis of variance (ANOVA) for between-group differences. Categorical variables were expressed as frequencies and percentages, and the distribution among groups was analyzed by the chi-square test or the Fisher’s exact test. We considered P-values of <0.05 to be statistically significant. Multiple logistics and linear regression analysis were used to adjust confounders. Different regression models were adjusted for different confounder combinations (see Results). All confounders adjusted in multiple regression analysis for obstetric and perinatal parameters fertilization methods, stage of the embryo, fertilization rate, number of embryos transferred, endometrial thickness before transplanting, type of infertility, weight gain during pregnancy,parity,preterm birth, fetal gender, birth weight, gestational diabetes mellitus, (GDM), and PIH hypertensive disorder of pregnancy (HDP). All confounders were adjusted in multiple regression analysis for the height, weight, and BMI of children, including gender, age, weight gain during pregnancy, parity, endometrial thickness, before transplanting, fertilization methods, stage of the embryo, fertilization rate, type of infertility, number of embryos transferred, breastfeeding, GDM, HDP, preterm birth socio-economic status (highest education, job occupation, and income per month), maternal height, and maternal weight.

## Results

There were 6,705 patients enrolled in this study. Among these, 745 patients were in the DFT group, and 2,980 patients were enrolled separately in FET and ET groups (**Table 1**). Women in the DFT group gained the least weight compared with ET group and FET group. More women in the ET group were experiencing their first delivery than in the FET and DFT groups. The type of infertility and cause of infertility in the DFT group were different to the other two groups. More blastocyst transfers were carried out in the DFT and FET groups (96.4% and 96.7%, respectively) than in the ET group (24.0%; **Table 1**).

**Table 1:** Demographic and clinical characteristics of the patients at the baseline.

	DFT (N = 745)	FET (N = 2,980)	ETs (N = 2,980)	P-value
Maternal characters				
Maternal age (y)	30.8 ± 4.0	30.8 ± 3.9	31.0 ± 3.9	0.134
BMI (kg/m^2^)	23.8 ± 3.9	23.8 ± 3.8	23.6 ± 3.8	0.252
Weight gain during pregnancy (kg)	13.9 ± 8.0	14.9 ± 7.6	14.9 ± 7.0	0.003*
Parity n (%)				0.035*
First	600 (80.5)	2464 (82.7)	2529 (84.9)	
Second	141 (18.9)	501 (16.8)	442 (14.8)	
Third or more	4 (0.5)	15 (0.5)	9 (0.3)	
Type of infertility n (%)				<0.001*
Primary infertility	589 (79.1)	1774 (59.5)	1664(55.8)	
Secondary infertility	156 (20.9)	1206 (40.5)	1316 (44.2)	
Cause of infertility n (%)				
Male factor	717 (96.2)	1536 (51.5)	2588 (86.8)	<0.001*
Ovulation disorder	115 (15.4)	718 (24.1)	481 (16.1)	<0.001*
Tubal factor	561 (75.3)	2222 (74.6)	2312 (77.6)	0.022*
Endometriosis	57 (7.7)	108 (3.6)	111 (3.7)	<0.001*
Unexplained	1 (0.1)	33 (1.1)	28 (0.9)	<0.001*
Others	363 (49.4)	122 (4.1)	64 (2.1)	<0.001*
Embryo characters n (%)				
No. of embryos transferred	1.1 ± 0.4	1.2 ± 0.4	1.7 ± 0.5	<0.001*
Endometrial thickness (cm)	1.0 ± 0.2	1.0 ± 0.2	1.1 ± 0.2	<0.001*
Fertilization rate	551 (93.4)	2400 (95.0)	2898 (97.3)	<0.001*
Stage at ET (%)				<0.001*
Cleavage	27 (3.6)	98 (3.3)	2264 (76.0)	
Blastocyst	716 (96.4)	2882 (96.7)	716 (24.0)	
Fertilization methods (%)				<0.001*
IVF	657 (88.2)	1878 (63.0)	2130 (71.5)	
ICSI	77 (10.3)	834 (28.0)	826 (27.7)	
PGT	11 (1.5)	268 (9.0)	24 (0.8)	

Presented as n (%) for categoric variables and mean ± SD for continuous variables. DFT, double frozen embryo transfer; FET, single frozen embryo transfer; ET, fresh embryo transfer; BMI, body mass index; IVF, in vitro fertilization; ICSI, intracytoplasmic sperm injection; PGT, preimplantation genetic diagnosis.*means that the p value was statistically significant.

**Table 2** presents perinatal and neonatal outcomes. The proportion of males born in the DFT group was higher than in the ET group (56.9% vs. 51.1%). The birth weight in both DFT and FET groups were heavier than that for ET (3462 g vs. 3412 g; 3458 g vs. 3412 g). The birth weight and risk of LGA tend to increase as the times of freezing increased (*P*-value for trend <0.001). The FET group had the lowest rate of vaginal delivery (27.2%) compared with DFT (31.8%) and ET (39%). The ratio of LGA was highest in the DFT group, and the FET group showed a higher LGA ratio than the ET group. Both neonatal disease and birth defect ratio were highest in the FET group.

**Table 2:** Obstetric and neonatal outcomes.

Variable	DFT (N = 745)	FET (N = 2980)	ET (N = 2980)	P-value	DFT vs. FET	DFT vs. ET	FET vs. ET
Gestational age (week)	39.17 ± 1.75	39.10 ± 1.71	39.23 ± 1.60	0.011*	0.302	0.381	0.003*
Gender				0.014*	0.069	0.005*	0.126
Male (%)	424 (56.9)	1583 (53.1)	1523(51.1)				
Female (%)	321 (43.1)	1397 (46.9)	1457 (48.9)				
Birth weight (g)	3462.4 ± 530.5	3457.9 ± 534.3	3411.6 ± 517.4	0.001*	0.834	0.019	0.001*
Weight							
≤1500	7(0.9)	22(0.7)	13(0.4)	0.173	–	–	–
1500–2500	19 (2.6)	99 (3.3)	103 (3.5)	0.462	–	–	–
4000–4500	96 (12.9)	372 (12.5)	343 (11.5)	0.403	–	–	–
≥4500	15 (2.0)	73 (2.4)	53 (1.8)	0.193	–	–	–
Body length (cm)	50.3 ± 2.1	50.3 ± 2.1	50.3 ± 1.9	0.934	–	–	–
Mode of				<0.001*	0.013*	<0.001*	<0.001*
Vaginal (%)	237 (31.8)	811 (27.2)	1162 (39.0)				
Cesarean (%)	508 (68.2)	2,169 (72.8)	1818 (61.0)				
GDM (%)	45 (6.0)	244 (8.2)	238 (8.0)	0.141	–	–	–
HDP (%)	44 (5.9)	204 (6.8)	124 (4.2)	<0.001*	0.357	0.040	<0.001*
Breastfeeding (%)	722 (98.1)	2,894 (98.1)	2885 (97.9)	0.914	–	–	–
SGA (%)	34 (4.6)	115 (3.9)	150 (5.0)	0.089	–	–	–
LGA (%)	230 (30.9)	877 (29.4)	240 (24.8)	<0.001*	0.446	0.001*	<0.001*
Preterm birth (%)	47 (6.3)	204 (6.8)	186 (6.2)	0.621	–	–	–
Perinatal death (%)	1 (0.1)	2 (0.1)	1 (0.0)	0.588	–	–	–
Neonatal mortality (%)	1 (0.1)	2 (0.1)	2 (0.1)	0.819	–	–	–
Polyhydramnios (%)	2 (0.3)	21 (0.7)	16 (0.5)	0.362	-	-	-
Oligohydramnios (%)	32 (4.3)	132 (4.4)	97 (3.3)	0.054	-	-	-
Placental deformity (%)	13 (1.7)	73 (2.4)	67 (2.2)	0.514	–	–	–
Placenta implantation (%)	1 (0.1)	8 (0.3)	3 (0.1)	0.345			
Placenta previa (%)	2 (0.3)	12 (0.4)	8 (0.3)	0.721	–	–	–
Placental abruption (%)	1 (0.1)	2 (0.1)	4 (0.1)	0.640	–		
Abnormal umbilical cord (%)	146 (0.3)	623 (20.9)	571 (19.2)	0.233	-	-	-
Neonatal disease (%)	18 (2.4)	143 (4.8)	84 (2.8)	<0.001*	0.005*	0.617	<0.001*
Birth defects (%)	13 (1.7)	82 (2.8)	33 (1.1)	<0.001*	0.152	0.192	<0.001*

DFT, double frozen embryo transfer; FET, single frozen embryo transfer; ET, fresh embryo transfer; SGA, small for gestational age; LGA. large for gestational age; NICU, neonatal intensive care unit; GDM, gestational diabetes mellitus; HDP, hypertensive disorder of pregnancy; *means p value was statistically significant.

**Tables 3** and **4** show the multiple regression analysis for obstetrics and neonatal and child development. The ET group was set as the reference. After adjustment for different confounder combinations in the three models, the difference of birth weight, LGA, and neonatal diseases was still significant among groups (**Table 3**). In multiple linear regression analysis of child development, the height Z-score of children born from the DFT and FET groups was higher than that for children from the ET group (β = 0.21, 95% CI 0.07–0.35; b = 0.17, 95% CI 0.05–0.28, respectively). Increasing height was also associated with times of freezing procedures increased ((P-value for trend = 0.003). However, body weight Z-score and BMI Z-score were not significantly different among the three groups (**Table 4**).

**Table 3:** Multivariate regression models for obstetrics and neonatal outcomes.

	Model 1 (OR/β, 95% CI)	Model 2 (OR/β, 95% CI)	Model 3 (OR/β, 95% CI)
Birth weight			
ET	Ref.	Ref.	Ref.
FET	**61.77 (18.70, 102.84)**	**61.28 (18.42, 104.13)**	**56.55 (19.99, 93.12)**
DFT	**84.11 (28.41, 139.81)**	**81.64 (26.22, 137.05)**	**72.22 (23.31, 121.12)**
P _trend_^b^	**<0.001**	**<0.001**	**0.002**
Gestational age			
ET	Ref.	Ref.	Ref.
FET	0.05 (−0.08, 0.19)	0.05 (−0.08, 0.19)	0 (−0.09, 0.09)
DFT	0.10 (−0.08, 0.27)	0.10 (−0.07, 0.28)	0.05 (−0.07, 0.17)
P _trend_^b^	0.27	0.24	0.48
Birth weight Z-score			
ET	Ref.	Ref.	Ref.
FET	**0.14 (0.05, 0.23)**	**0.14 (0.05, 0.23)**	**0.15 (0.06, 0.24)**
DFT	**0.18 (0.06, 0.30)**	**0.18 (0.06, 0.30)**	**0.18 (0.06, 0.30)**
P _trend_^b^	**0.002**	**0.002**	**0.001**
PIH			
ET	Ref.	Ref.	Ref.
FET	1.21 (0.82, 1.77)	1.21 (0.82, 1.77)	1.17 (0.79, 1.74)
DFT	1.21 (0.75, 1.97)	1.22 (0.75, 1.98)	1.21 (0.72, 2.03)
LGA			
ET	**Ref.**	**Ref.**	**Ref.**
FET	1.16 (0.99, 1.38)	1.16 (0.99, 1.38)	**1.28(1.07, 1.55)**)
DFT	**1.31 (1.07, 1.62)**	**1.31 (1.07, 1.62)**	**1.53(1.19, 1.97))**
P _trend_^b^	**<0.001**	**<0.001**	**0.001**
Birth defect			
ET	Ref.	Ref.	Ref.
FFT	1.98 (0.95, 4.12)	2.03 (0.97, 4.25)	**2.66 (1.25, 5.64)**)
DET	1.63 (0.68, 3.91)	1.67 (0.69, 4.07)	1.97 (0.77, 5.03)
Neonatal disease			
ET	Ref.	**Ref.**	**Ref.**
FFT	**2.41 (1.46, 3.96)**	**2.41 (1.46, 3.98)**	**2.77 (1.62, 4.74)**
DET	0.87 (0.43, 1.76)	0.87 (0.43, 1.78)	1.29 (0.60, 2.76)

Model 1: weight gain during pregnancy, parity, type of infertility, male factor, ovulation disorder, tubal factor, endometriosis, unexplained, fertilization rate, stage at ET, fertilizationmethods, no. of embryos transferred, Endometrial thickness. Model 2: model 1 + fetal gender. Model 3: model 1 + GDM + HDP + fetal gender + premature birth. Bold numbers indicate statistical significance (P < 0.05)： P trend^b^ means p for trend.

**Table 4:** Height, weight, and BMI z scores and their coefficients and 95% CIs from unadjusted and adjusted regression models.

	Unadjusted (β, 95% CI)	Model 1 (β, 95% CI)	Model 2 (β, 95% CI)	Model 3 (β, 95% CI)
Height Z-score				
ET	Ref.	Ref.	Ref.	Ref.
FET	0.01 (**−**0.07, 0.08)	**0.12 (0.00, 0.24)**	**0.09 (0.02, 0.16)**	**0.17 (0.05, 0.28)**
DFT	0.10 (**−**0.02, 0.21)	**0.15 (0.00, 0.29)**	**0.20 (0.08, 0.31)**	**0.21 (0.07, 0.35)**
P _trend_^b^	–	**0.048**	**0.001**	**0.003**
Weight Z-score				
ET	Ref.	Ref.	Ref.	Ref.
FET	****−**0.14 (**−**0.27, **−**0.00)**	**−** 0.04 (**−** 0.25, 0.18)	****−**0.21 (**−**0.35, **−**0.07)**	**−** 0.12 (**−** 0.33, 0.11)
DFT	**−** 0.05 (**−** 0.26, 0.17)	0.06 (**−** 0.22, 0.33)	0.08 (**−** 0.14, 0.30)	0.14 (**−** 0.14, 0.41)
P _trend_^b^	–	0.74	0.42	0.42
BMI Z-score				
ET	Ref.	Ref.	Ref.	Ref.
FET	0.07 (**−** 0.02, 0.16)	0.04 (**−** 0.10, 0.18)	**−** 0.03 (**−** 0.12, 0.06)	**−** 0.05 (**−** 0.19, 0.10)
DFT	0.02 (**−** 0.12, 0.17)	0.06 (**−** 0.12, 0.24)	0.08 (**−** 0.06, 0.23)	0.08 (**−** 0.10, 0.27)
P _trend_^b^	–	0.56	0.54	0.44

Ref., Reference; Model 1: adjusted for weight gain during pregnancy, parity, endometrial thickness before transplanting, fertilization methods, stage of the embryo, fertilization rate, number of embryos transferred, type of infertility, male factor, ovulation disorder, tubal factor, endometriosis, and unexplained. Model 2: weight gain during pregnancy, breastfeeding, GDM, the highest education, income in a month,PIH, occupation, maternal height (height Z-score), maternal weight (weight Z-score), or maternal BMI (BMI Z-score). Model 3: model 1 + model 2 + premature birth. Bold numbers indicate statistical significance (P < 0.05). Data were analyzed by multiple mixed linear model through R.4.0.

For the high ratio of donor sperm in the DFT group, a subgroup analysis, excluding the male partners age >35 years and with severe sperm deficiency or azoospermia, was carried out. The subgroup outcomes were consistent with overall outcomes (see **Supplementary Tables 1** and **2**).

## Discussion

This nested case-control study included 6,705 women who had a singleton live birth after embryo transplant. We found that the birth weight and LGA proportion in the DFT and FET groups were significantly higher compared with that in the ET group. In addition, the test for trend showed that the birth weight and risk of LGA tended to increase as the times of freezing increased. In the comparison of children’s development, the height Z-score of children in the DFT group was greater than in the ET group and the trend test also was significant. However, there was no significant difference in body weight and BMI Z-scores of children born from DFT group than that from FET and ET group after adjustment.

Embryo cryopreservation methods especially for blastocysts have changed from slow freezing to vitrification according to safety and efficacy of the reports over the past decade (16–18). Vitrification is an ultrarapid cryopreservation method with a high concentration of permeable cryoprotectants, which have raised concerns about possible “toxicity” to the embryos and even to the offspring (19). Studies have observed reduced risks of preterm birth and low birth weight in FET cycles compared with that in fresh ETs (6). However, in a large cumulative meta-analysis, singletons born after FET were found to have an increased risk of being born LGA and having a heavier birth weight; there was also an increased risk of HDP (4, 5). We demonstrated similar effects in the present study. The birth weight and LGA rate in the DFT and FET groups were both significantly higher than that in ET group. After adjusting confounders by multiple regression analysis, we found that the birth weight was still higher in the FET and DFT groups. In terms of LGA rate, there was still a significant difference between the DFT and ET groups after adjustment, but the significance was no longer present in a comparison between FET and ET group. Moreover, it is important to realize that there was an increased trend among three groups in birth weight, birth weight Z-score, and LGA rates (showed by the *P*-values for trend), when the ET group was set as the reference in multiple regression analysis. This situation continued in the multiple regression analysis of height Z-score in the results of child development. All these outcomes demonstrated a cumulative effect of gamete cryopreservation and embryo cryopreservation.

Several pathophysiological processes may play roles in the low risk of SGA and higher risk of LGA in FET cycles than that in fresh ET cycles. The first one is that increased hormone blood levels, especially high estrogen levels, might alter the timing of endometrial receptivity and exert a detrimental effect on spiral artery remodeling by the trophoblast (20, 21). A potential role in placental function dysregulation for elevated estrogen exposure has been associated with higher rates of low birth weight and fetal growth restriction. (22). The second explanation proposed for the increased risk of LGA with frozen cycles is the epigenetic changes during freezing and thawing. The cryopreservation technique may cause epigenetic changes within the embryos, such as DNA methylation and histone modification (23, 24). In the present study, DFT and FET group were mostly at the blastocyst stage. It has also been shown that higher birth weight, and higher risk of LGA and VLGA are found in blastocyst vs. cleavage stage transfer, which is related to the greater number of epigenetic changes during extended culture (25–27). However, the variable of embryo stage at transfer was adjusted by multiple regression analysis, and the higher risk of LGA still existed in the DFT group compared with that in the ET group. Therefore, DFT group showed an increased trend in birth weight and LGA rate compared with FET group; this might possibly be related to epigenetic changes, as the DFT group had all the same parameters as FET group except for one additional gamete cryopreservation procedure. The freezing and thawing procedures performed in gametes and embryo stages might induce cumulus epigenetic changes and stress reactions. Moreover, the results of the long-term follow-up supported the theory that an epigenetic programming of metabolism during prenatal and postnatal periods, as a response to imprinting alterations, occurred during early embryonic development (28, 29).

However, as we mentioned previously, most studies show no increased risk of adverse perinatal outcome following the use of cryopreserved sperm or oocytes. In the present study, DFT group mostly involved cryotransfer of embryos from cryopreserved donor sperm. So why does gamete cryopreservation alone not exhibit an influence on perinatal outcomes, whereas the combination of gametes and embryo cryopreservation shows different outcomes from embryo cryopreservation alone? There might be a threshold for the epigenetic changes or the remodeling of epigenetics during meiosis and early embryo development (30) that covers the epigenetic changes during gamete freezing and thawing. The clear etiological and pathophysiological mechanisms need to be revealed.

Some studies related to double frozen procedures include repeated cryopreservation of embryos. One situation when this may take place is when a surplus of zygotes or day 3 embryos are warmed and cultured for blastocyst development (31, 32). When more blastocysts are formed than required for transfer, repeated cryopreservation may be considered. Another scenario is the repeated vitrification and warming of blastocysts for preimplantation genetic diagnosis (PGD) (33); in such repeated embryo cryopreservation, the clinical pregnancy rate and live birth rate were found to be decreased (31–33). However, limited data regarding perinatal outcomes and long-term follow up have been reported.

Interpretation of associations from observational studies is always challenging. Although we conducted a strict nested case-control study with a large sample size and adjusted for many confounders, several limitations of this study should still be noted. First, most gamete cryopreservation was of donor frozen sperm. As sperm donors are relatively young and have normal semen, there was a selection bias in the DFT population. Therefore, a subgroup analysis, excluding those male partners age >35 years and with severe sperm deficiency or azoospermia, was carried out, and the subgroup outcomes were consistent with the overall findings. Second, not all confounders were taken into accounts, owing to the retrospective nature of this study. Third, all children conceived by ART in this study were from a single medical center in Shandong, China; therefore, caution should be taken in generalizing these findings.

## Conclusions and Perspectives

In conclusion, there is an increased risk of LGA babies associated with pregnancies conceived from DFT. Furthermore, the children are inclined to be taller in the future in this group compared with offspring following FET. The related etiology and pathophysiology mechanisms still need to be revealed. In the future, well-designed, observational studies with an in-depth collection of patient characteristics may shed more light on this issue.

## Data Availability Statement

The raw data supporting the conclusions of this article will be made available by the authors, without undue reservation.

## Ethics Statement

The studies involving human participants were reviewed and approved by the Institutional Review Board of the Second Hospital, Cheeloo College of Medicine, Shandong University. The patients/participants provided their written informed consent to participate in this study.

## Author Contributions

SG and LC contributed to the study concept and design. JG, YZ, and BW analyzed data and drafted the paper. TZ, LC, and Z-JC contributed to the review and the revision of the manuscript. All authors contributed to the article and approved the submitted version.

## Funding

The National Natural Science Foundation of China (82171692), Research Unit of Gametogenesis and Health of ART-Offspring, Chinese Academy of Medical Sciences (2020RU001), Taishan Scholars Program for Young Experts of Shandong Province (tsqn201909195), and Natural Science Foundation of Shandong Province (ZR2021QH136).

## Conflict of Interest

The authors declare that the research was conducted in the absence of any commercial or financial relationships that could be construed as a potential conflict of interest.

## Publisher’s Note

All claims expressed in this article are solely those of the authors and do not necessarily represent those of their affiliated organizations, or those of the publisher, the editors and the reviewers. Any product that may be evaluated in this article, or claim that may be made by its manufacturer, is not guaranteed or endorsed by the publisher.

## Supplementary Material

Supplementary Figure 1 Flow Chart. DFT, double frozen embryo transfer; FET, single frozen embryo transfer; ET, fresh embryo transfer.

Supplementary Table 1 Sensitivity analysis of obstetrics and neonatal outcomes. DFT, double frozen embryo transfer; FET, single frozen embryo transfer; ET, fresh embryo transfer; LGA, large for gestational age; Ref., reference. weight gain during pregnancy, parity, type of infertility, male factors, ovulation disorder, tubal factor, endometriosis, unexplained, fertilization rate, stage at ET, fertilization methods, no. of embryos transferred, Endometrial thickness, GDM, HDP, fetal gender, and premature birth. (P < 0.05).

Supplementary Table 2 Sensitivity analysis of children development. DFT, double frozen embryo transfer; FET, single frozen embryo transfer; ET, fresh embryo transfer; Ref., reference. weight gain during pregnancy, parity, endometrial thickness before transplanting, fertilization methods, stage of the embryo, embryo fertilization level, no. of embryos transferred, type of infertility, male factor, ovulation disorder, tubal factor, endometriosis, unexplained, breastfeeding, GDM, the highest education, income in a month, occupation, maternal height (height Z-score), maternal weight (weight Z-score), or maternal BMI (BMI Z-score), and premature birth.
