# Supplementary material for: "Double Frozen Transfer" Could Influence the Perinatal and Children's Growth: A Nested Case-Control Study of 6705 Live Birth Cycles
Source: Front Endocrinol (Lausanne). 2022 Aug 12;13:878929. doi: 10.3389/fendo.2022.878929 (PMC9413401; doi:10.3389/fendo.2022.878929)
Supplement: Supplementary Figure 1 — Flow Chart. DFT, double frozen embryo transfer; FET, single frozen embryo transfer; ET, fresh embryo transfer. [file Table_1.doc]

**Supplementary table 1 birth defect according to ICD**

| **Variable** | **DFTs**  **(N=745)** | **FETs**  **（N=2980）** | **ETs**  **（N=2980）** | **P Value** | **DFTs**  **VS**  **FETs** | **DFTs**  **VS**  **ETs** | **FETs**  **VS**  **ETs** |
| --- | --- | --- | --- | --- | --- | --- | --- |
| **Nervous system malformation ICD,10 codes Q00–07 $** | 0（0） | 2（0.1） | 8（0.3） | 0.115 | - | - | **-** |
| **Congenital malformation of eye, ear, face and neck, ICD,10 codes Q10–18 $** | 4（0.5） | 17（0.6） | 1（0.0） | 0.002 | 0.770 | 0.307 | 0.307 |
| **Cardiovascular malformation, ICD,10 codes Q20–28 $** | 4（0.5） | 14（0.5） | 9（0.3） | 0.597 | - | - | **-** |
| **Respiratory malformation, ICD,10 codes Q30,34 $** | 2（0.3） | 10（0.3） | 3（0.1） | 0.194 | - | - | **-** |
| **Cleft palate/lip, ICD,10 codes Q35–37 $** | 0（0.0） | 3（0.1） | 2（0.1） | 1.000 | - | - | **-** |
| **Gastrointestinal malformation,**  **ICD,10 codes Q40–43 $** | 1（0.1） | **8（0.3）** | 0（0.0） | 0.008 | 0.698 | 0.200 | 0.008 |
| **Genital organs malformation,**  **ICD,10 codes Q50–56 $** | 0（0.0） | 3（0.1） | 0（0.0） | 0.341 | - | - | **-** |
| **Urogenital malformation,**  **ICD,10 codes Q60–64 $** | 1（0.1） | 11（0.4） | 3（0.1） | 0.083 | - | - | **-** |
| **Musculoskeletal malformation,**  **ICD,10 codes Q65–79 $** | 1（0.1） | **14（0.5）** | 4（0.1） | 0.043 | 0.330 | 1.000 | **0.031** |
| **Chromosomal malformation,**  **ICD,10 codes Q90–99 $** | 0（0.0） | 2（0.1） | 0（0.0） | 0.605 | - | - | **-** |
| **Other** | 4（0.5） | 15（0.5） | 4（0.1） | 0.032 | 1.000 | 0.056 | **0.019** |
| **Note: Other including congenital metabolic diseases, hernia and abnormal organ position and so on . *means P<0.05; ICD,10=International Classification of Diseases, 10th edition.** | | | | | | | |

|  | **DFTs** | **FETs** | **ETs** | **P value** | **DFTs VS FETs** | **DFTs VS ETs** | **FETs VS ETs** |
| --- | --- | --- | --- | --- | --- | --- | --- |
| **Number of visits** | 862 | 3821 | 4738 |  |  |  |  |
| **Age-year** | 1.68±1.40 | 1.61±1.38 | 1.37±1.94 | <0.001* | 0.188 | <0.001* | <0.001* |
| **Sex** |  |  |  | <0.001* |  |  |  |
| Male | 479(55.4) | 2055(41.9) | 2373(48.4) |  |  |  |  |
| Female | 386(44.6) | 1770(39.0) | 2377(52.4) |  |  |  |  |
| **Height-cm** | 84.51±14.57 | 83.56±14.59 | 82.47±11.99 | 0.009 | 0.118 | <0.001* | 0.002* |
| **Height Z-score** | 0.84±1.07 | 0.75±1.12 | 0.72±1.13 | 0.283 | - | - | - |
| **Weight-kg** | 12.65±5.25 | 12.32±5.35 | 11.70±3.47 | <0.001* | 0.129 | <0.001* | <0.001* |
| **Weight Z-score** | 1.37±2.42 | 1.21±2.05 | 1.40±2.59 | <0.001* | 0.063 | 0.780 | 0.001* |
| **BMI-kg/cm2** | 17.43± 2.03 | 17.46±2.18 | 17.45±2.08 | 0.337 | - | - | - |
| **BMI Z-score** | 0.65±1.22 | 0.67±1.36 | 0.62±1.27 | 0.159 | - | - | - |

**Supplementary table 2 Mean and SD of height, BMI and their z score.**

**Supplementary table 3**

|  | **Excluded participants with severe disease of male factor and age>35** | **All participants** |
| --- | --- | --- |
| **Birth weight** |  |  |
| ETs | **Ref.** | Ref. |
| FETs | **66.14(15.39,116.89)** | **61.77(18.70, 102.84)** |
| DFTs | **101.51(39.76, 163.25)** | **84.11(28.41, 139.81)** |
| **Gestational age** |  |  |
| ETs | **Ref.** | Ref. |
| FETs | 0.03 (-0.13,0.19) | 0.05（-0.08，0.19） |
| DFTs | 0.12(-0.08,0.31) | 0.10（-0.08，0.27） |
| **Birth weight Z-Score** |  |  |
| ETs  FETs  DFTs | **Ref.**  **0.17(0.06, 0.28)**  **0.22(0.09, 0.35)** | **Ref.**  **0.14（0.05，0.23）**  **0.18（0.06，0.30）** |
| **PIH** |  |  |
| ETs | Ref. | Ref. |
| FETs | **1.89 (1.11，3.22)** | 1.21（0.82，1.77） |
| DFTs | 1.48 (0.80，2.73) | 1.21（0.75，1.97） |
| **LGA** |  |  |
| ETs | **Ref.** | **Ref.** |
| FETs | **1.25(1.02，1.53)** | 1.16（0.99，1.38） |
| DFTs | **1.50(1.18，1.90)** | **1.31（1.07，1.62）** |
| **Birth defect** |  |  |
| ETs | **Ref.** | Ref. |
| FETs | 1.44(0.65,3.21) | 1.98（0.95，4.12） |
| DFTs | 1.06(0.41,2.79) | 1.63（0.68，3.91） |
| **Neonatal disease** |  |  |
| ETs | **Ref.** | Ref. |
| FETs | **2.52(1.35，4.68)** | **2.41（1.46，3.96）** |
| DFTs | 0.95(0.42，2.11) | 0.87（0.43，1.76） |

**Sensitivity analysis excluded male partners with age > 35yeare and with severe sperm deficiency or** [**azoospermatism**](javascript:;) **in perinatal and neonatal outcomes**

**Note: Severe disease of male factor including oligospermia, asthenozoospermia, teratozoospermia, non-obstructive azoospermia and so on**

|  | **Excluded participants with severe disease of male factor and age>35** | | | | | **All participants** | | | | |
| --- | --- | --- | --- | --- | --- | --- | --- | --- | --- | --- |
|  | | **unadjusted** | **1** | **2** | **3** | | **unadjusted** | **1** | **2** | **3** |
| **Height Z-score** | |  |  |  |  | |  |  |  |  |
| **ETs** | | **Ref.** | **Ref.** | **Ref.** | **Ref.** | | **Ref.** | **Ref.** | **Ref.** | **Ref.** |
| **FETs** | | 0.04(-0.04, 0.13) | **0.18(0.05,0.32)** | **0.12(0.03, 0.20)** | **0.24(0.11, 0.38)** | | **0.01(-0.07，0.08）** | **0.12(0.00，0.24）** | **0.09(0.02,0.16)** | **0.18(0.07，0.30)** |
| **DFTs** | | 0.08(-0.04,0.21) | 0.12(-0.04, 0.29) | **0.17(0.05, 0.30)** | **0.22(0.06,0.38)** | | **0.10(-0.02，0.21)** | **0.15(0.00，0.29)** | **0.20(0.08,0.31)** | **0.24(0.10，0.39)** |
| **Weight Z-score** | |  |  |  |  | |  |  |  |  |
| **ETs** | | **Ref.** | **Ref.** | **Ref.** | **Ref.** | | **Ref.** | **Ref.** | **Ref.** | **Ref.** |
| **FETs** | | **-0.10(-0.26,0.07)** | **0(-0.26,0.26)** | **-0.16(-0.33,0.01)** | **-0.09(-0.35,0.18)** | | **-0.14(-0.27，-0.00）** | **-0.04(-0.25，0.18)** | **-0.21(-0.35, -0.07)** | **-0.11(-0.33，0.11)** |
| **DFTs** | | **-0.08(-0.32, 0.17)** | **0.02(-0.29,0.33)** | **0.05(-0.19,0.30)** | **0.11(-0.21, 0.42)** | | **-0.05（-0.26，0.17）** | **0.06(-0.22，0.33）** | **0.08(-0.14，0.30)** | **0.15(-0.13，0.42）** |
| **BMI Z-score** | |  |  |  |  | |  |  |  |  |
| **ETs** | | **Ref.** | **Ref.** | **Ref.** | **Ref.** | | **Ref.** | **Ref.** | **Ref.** | **Ref.** |
| **FETs** | | **0.09(-0.02,0.20)** | **0.10(-0.06, 0.27)** | **0(-0.12,0.11)** | **0.01(-0.16, 0.18)** | | **0.07(-0.02，0.16)** | **0.04(-0.10，0.18)** | **-0.03(-0.12，0.06)** | **-0.04(-0.19，0.10)** |
| **DFTs** | | **0(-0.16,0.16)** | **0.07(-0.13, 0.27)** | **0.05(-0.12, 0.21)** | **0.08(-0.12,0.29)** | | **0.02(-0.12，0.17)** | **0.06(-0.12，0.24)** | **0.08(-0.06，0.23)** | **0.09(-0.09，0.27)** |

**Supplementary table 4**

**Sensitivity analysis in children development excluded male partners with age > 35yeare and with severe sperm deficiency or** [**azoospermatism**](javascript:;)
